# Brokering peace in the ape (culture) wars

**DOI:** 10.1017/ehs.2026.10039

**Published:** 2026-03-06

**Authors:** Ammie K. Kalan, Claudio Tennie

**Affiliations:** 1 GAB Lab, Department of Anthropology, University of Victoria, Victoria BC, Canada; 2 DFG Center for Advanced Studies, ‘Words, Bones, Genes, Tools’, University of Tubingen, Tubingen, Germany; 3 Early Prehistory and Quaternary Ecology, Faculty of Science, University of Tubingen, Tubingen, Germany; 4 Lester E. Fisher Center for the Study and Conservation of Apes, Lincoln Park Zoo, Chicago, IL, USA

**Keywords:** field research, comparative cognition, primatology, animal cultures, scientific bias

## Abstract

Mid-last century, controversy existed around the question whether non-human great apes have culture. To a large degree, this is no longer controversial – apes have their own cultures. However, there remains controversy around how to best study ape culture, given the varying and often dichotomised contributions from captive and field-ape research. Here, we present a historical summary of the ape culture wars since their inception and how this has evolved over time. We then focus on debates surrounding wild versus captive-ape research with an emphasis on culture, detailing major arguments arising from both research domains. Throughout, we critically deconstruct these arguments, illustrating the nuance behind these critiques, while highlighting their assumptions, overgeneralising statements and potential constraints. We further provide potential solutions to help alleviate the issues we describe, when possible. We also offer a metacritique of ape culture research for its scientific and political impact, irrespective of one’s expertise. In closing, we summarise concrete recommendations for a richer and more holistic understanding of ape, and human, culture.

## Graphical abstract

**Figure:**
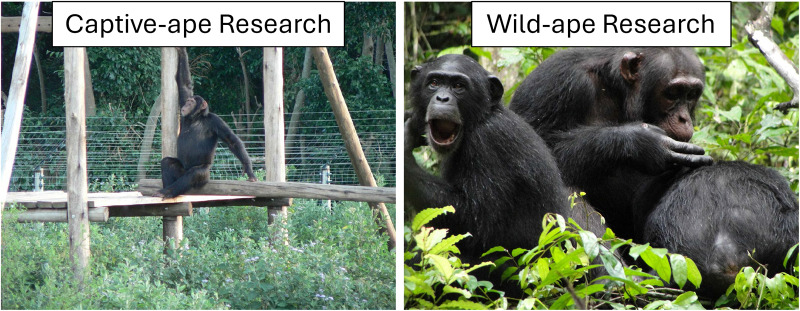

## 1. Introduction: what are the ape culture wars?

**‘Culture is a fighting word**’ Langlitz, N. 2020, p. 55 from *Chimpanzee Culture Wars*

The ‘ape culture wars’ is a term used to designate the controversy around attributing human-like culture to great apes. Originally coined specifically for chimpanzees by primatologist William McGrew (McGrew, 2003), the term was fitting for the debates that followed, especially after the first publications of cross-population behavioural differences described as culture in wild chimpanzees (McGrew & Tutin, 1978; McGrew et al., 1979; Whiten et al., 1999, 2001). These debates came to be known as ‘the ape culture wars’ (also referred to as the ‘chimpanzee culture wars’ (Langlitz, 2020), but as all apes have culture, we prefer the general term). From a social perspective, captive-ape researchers, often equated with psychologists, were perceived as being *against* the existence of human-like ape cultures, while wild-ape researchers, often equated with primatologists or biologists generally, were perceived as being *for* the existence of such ape cultures. While there are exceptions to these designations, a deeper look into the divide between wild and captive-ape research is key to a better understanding of – and, with it, potentially overcoming – the ape culture wars. Broadly speaking, this kind of ‘war’ is not unique to ape culture, rather it appears in other interdisciplinary topics where what could separate human from nonhuman is investigated, such as cooperation, altruism, reciprocity, etc. These kinds of culture wars relate to scientific cultures. Here, we concentrate on the ape *culture* war, with special emphasis on the most-often-contrasted sides: wild- vs. captive-ape research.

Another aspect within the ape culture wars relates to the question of what culture is. While several definitions have been proposed over the years (see Langlitz, 2020 for a historical review), there are heated debates around what type(s) of social learning are acceptable for specific types of culture (e.g. imitation; know-how copying), as well as what types of cultural products are acceptable (e.g. norms; multiple behaviours affected; temporal persistence) and under what circumstances (e.g. conformity; joint attention). The aim of this piece is not to resolve these complicated issues. Indeed, the idea of doing so may be futile given details reasonably depend on one’s research question. In our view, what meaningfully counts as culture may simply have more to do with what kinds of questions one seeks to answer. If so, we might have to embrace a plurality of approaches and definitions. However, given how widespread different types of social learning are in the animal kingdom, there is widespread potential for various kinds of animal cultures.

According to most culture definitions, where there is social learning, there is potential – but not automatic proof – of culture. Evidence for the litmus test for social learning is sometimes indirect – where between-population behavioural differences cannot be explained (at least not fully) by genetic and/or environmental differences. Here, social learning can become the most parsimonious explanation. This approach is also known as the method of exclusion (Krützen et al., 2007), or the ethnographic method (Laland & Janik, 2006). Since the initial descriptions of wild chimpanzee culture using this method (Whiten et al., 1999, 2001), culture has now been similarly documented in orangutans, bonobos and to some extent gorillas. Hence, all great apes show this indirect evidence for social learning (*chimpanzees*: Badihi et al., 2023; Boesch & Boesch-Achermann, 2000; Boesch et al., 2020; Goodall, 1986; Kalan et al., 2020; Luncz et al., 2012; Matsuzawa et al., 2011; Nishida, 2011; van Leeuwen, Cronin, et al., 2014; van Leeuwen et al., 2012; Whiten et al., 1999, 2001; Wrangham et al., 1994; *bonobos*: Hohmann & Fruth, 2003; Samuni et al., 2022; *orangutans*: Krützen et al., 2011; Lameira et al., 2013; van Schaik et al., 2003; Wich et al., 2012; *gorillas*: Robbins et al., 2016; see also Kalan et al., 2023). Indirect evidence for social learning is also found in other primates, such as capuchins (Perry, 2011) and macaques (Leca et al., 2010; Luncz et al., 2019; Schofield et al., 2018). There is also direct evidence for social learning, via experiments or tracking of social transmission, in monkeys (van de van de Waal et al., 2013) and in apes – and, with it, culture (*chimpanzees*, e.g. Hobaiter et al., 2014; van Leeuwen & Hoppitt, 2023), including cases in which models pay a cost (i.e. teaching, Musgrave et al., 2020) – in *bonobos* (Shorland et al., 2019), *gorillas* (Stoinski et al., 2001), and *orangutans* (Stoinski & Whiten, 2003). Evidence for social learning and culture has also been documented for non-primates such as birds (Aplin, 2019), cetaceans (Whitehead & Rendell, 2015), reptiles (Kis et al., 2015), and insects (Bridges et al., 2024).

As mentioned above, some researchers insist that the term culture should be reserved for cases where particular types of social learning are present. Indeed, there were already discussions about the presence versus absence of specific social learning mechanisms in apes more than one hundred years ago (e.g., Yerkes vs. Köhler). Nevertheless, the resulting corpus of research on social learning, and the accepted general link between culture and social learning, succeeded in persuading academics, including captive-ape researchers, that animals do indeed have culture.

In some way, this could have meant an end to the ape culture war (in our personal view, circa 2005). Instead around the same time, the ‘war’ repositioned itself, given that humans are undeniably different in some way(s). This happened again with regards to the exact social learning type(s) responsible for cultures in different species – with explicit links to claims of special types of cultural products that may depend on special types of social learning. Regarding primates, captive-ape researchers tended to investigate how and why human culture is *different* than those of apes, while wild-ape researchers often dedicated their efforts to identifying ways in which ape culture is *similar* to human culture. These differing stances led to continued division, despite both groups now accepting that ape cultures of some types do exist.

The debate at hand has therefore evolved into a better understanding of the specifics of human and ape cultures. However, as is presented below, the field is plagued with misunderstandings and biases which in our view prevent the identification and appreciation of a surprising amount of (hidden) common ground. This has been the primary impetus for the current paper. As two colleagues from a place of mutual respect, although not complete agreement, we sought to write this paper in response to the divisive rhetoric we often see circulating in the field of ape culture research. Our differing expertise in wild-ape and captive-ape culture research are well aligned with the often-contrasting sides represented in the ape culture wars, creating an ideal situation for dialogue.

Overall, researchers working on ape culture have diverse backgrounds, methods, and research questions, working in diverse settings. In our view, this is a good thing. The study of ape culture thus far has immensely benefited from this diversity of captive and wild-ape research. Culturally relevant or adjacent aspects such as social learning, innovation, cognitive abilities, and tool use were first investigated in captive apes by psychologists in the early 1900s (Köhler, 1925; Yerkes, 1916). The first descriptions of suspected cultural tool use behaviour and potential cultural variation in wild apes appeared much later with the advent of long-term ape field research in the 1960s and 1970s (Goodall, 1963, 1964; McGrew & Tutin, 1978; McGrew et al., 1979; Nishida, 1973; Sugiyama & Koman, 1979). These research avenues continue, and they should be seen as complementary approaches, illuminating different sides of the same cultural coin. More specifically, wild-ape researchers mainly study the extent of naturally occurring cultural diversity, while captive-ape researchers mainly study the cognitive underpinnings generating and maintaining this cultural diversity. This dualism has continued to this day with sadly limited acknowledgement from either side.

Here, we first describe and deconstruct critiques often levied by wild-ape researchers against captive-ape researchers (Part B), and *vice versa* (Part C), to illustrate the nuance and problematic rationale of these critiques. Second, we examine critical issues affecting both captive and wild-ape research (Part D) with suggestions on how to improve current methods and best practices when possible (Part E and throughout). Our overall goal is for this paper to serve as an extended olive branch to researchers on both sides of the ape cultures debate.

## 2. Critiques from wild to captive-ape research

### 2.1. Lack of socio-ecological validity

**Figure 1.**
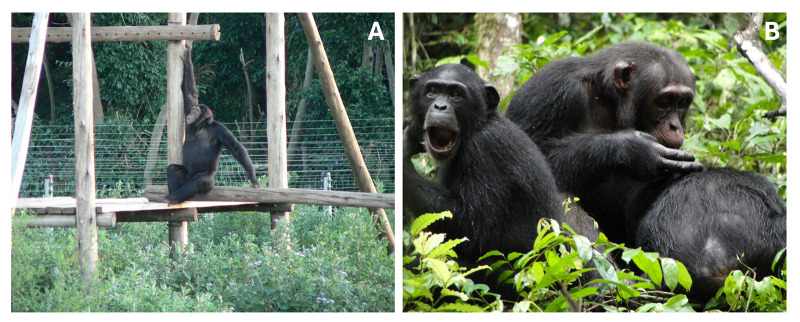
Photos of chimpanzees that have been part of ape culture research at (A) Ngamba Island Sanctuary in Uganda (*credit C. Tennie*) and (B) Taï Chimpanzee Project in Côte d’Ivoire (*credit A. Kalan*). Note the differences in environments.

Captive-ape studies in zoos or sanctuaries do not match the social and ecological environments apes naturally evolved in (Boesch, 2012, 2021; Whiten, 2022), also referred to as the ‘environment of evolutionary adaptedness’ or EEA (Bowlby 1969). The cognition of captively housed apes may as a result not be representative of their wild counterparts (Boesch, 2012, 2021). In the wild, apes typically live in much more varied, complex and less predictable environments, both socially and physically. They must use behavioural and cognitive strategies to forage flexibly, defend against predators or competitors, as well as fission and fuse with conspecifics (Aureli et al., 2008). Moreover, they face problems typically absent in captivity, such as toxic plants and seasonality. In contrast, captive settings are less varied and more predictable. They typically provide a socially and physically restricted setting, coupled with a safe environment and food stability, not comparable to the wild. In the not-so-distant past, captive ape settings frequently included extreme housing and treatment conditions such as asocial housing in tiny, barren cages, and/or invasive research procedures (Hosey, 2023). Moreover, in the not-so-distant past, captive apes were wild-sourced and today they continue to derive from wild populations in other ways (e.g. rescued from illegal trade). In all these cases, apes – nearly exclusively young apes – were removed from their mother’s care and their habitat; likely traumatising them. Importantly, early life experience can have effects on social and cognitive development which was recently corroborated in cognitive studies of captive apes (e.g. Bohn et al., 2023). Some captive apes originate from biomedical facilities or the entertainment industry where early-life trauma might affect socio-cognitive abilities (Clay & de Waal, 2013; van Leeuwen, Mulenga, et al., 2014). In sum, differences between captive-ape settings and the wild may result in immediate differences in behavioural expression but may also accumulate and solidify during ontogeny (Figure 1).

Where these differences exist, they might translate into detectable differences in social learning, cognition, and culture between captive and wild apes. We observe that wild-ape researchers sometimes apply this logic to all captive apes, assuming a cross-domain negative effect. However, variation in rearing history and trauma can also be present in wild apes, particularly in populations where hunting pressure or disease prevalence is high. In such wild situations, individuals may likewise endure trauma due to, for example, losing group members (in particular their mothers; Botero et al., 2013; Stanton et al., 2020). Recent research examining the long-term effects of being orphaned at a young age on behaviour, cognition and stress in both wild and sanctuary apes, suggests that this trauma has tangible effects for individuals in both environments (Botero et al., 2013; Clay & de Waal, 2013; Girard-Buttoz et al., 2021; Stanton et al., 2020; van Leeuwen, Mulenga, et al., 2014). Perhaps all or some of the effects of trauma can be overcome by time (Girard-Buttoz et al., 2021). What is currently empirically unresolved is how long the effects of various types of trauma last, and how much and how long cultural abilities may be affected.

Further complicating matters, captive apes are sometimes raised or trained by humans, in addition to interacting with humans quite regularly. This, too, may affect cultural cognition, both directly and indirectly. The typical outcome of such interactions seems to be positive effects on social learning and cognition. For example, early life experience with humans increases curiosity and exploration tendencies in rehabilitated wild and captive orangutans (Damerius et al., 2017; Forss et al., 2015). Enculturation is the now-established term for raising captive apes in human environments as if they are human, even though the correct term would be acculturation (more precisely: human acculturation). Captive apes have also been successfully trained to imitate, in so-called ‘Do-as-I-do’ studies, with brain scans taken before and after training showing relevant structural changes that matched the increased proficiency at the behavioural level (Pope et al., 2018). However, the degree of training and enculturation intersects, and is likely influenced by the captive environment and enrichment opportunities, for its influence on cognition. A recent study examining peering rates in orangutans, a behaviour researchers score to assess individuals’ attentiveness toward conspecifics as a precondition for social learning (Schuppli et al., 2016), found zoo-housed apes peered more than their wild counterparts (Kukofka et al., 2025). One potential explanation for this is the documented increased sociability in captive orangutans relative to wild populations (Forss et al., 2015; Kukofka et al., 2025). While enculturated and imitation-trained apes are rare and identifiable, it is difficult to disentangle all possible influences of human interaction from captive ape cognition. This situation is a potential validity threat to using captive ape performance as an estimate for truly wild ape cognition, i.e. uninfluenced by human interaction and contact. However, wild ape data are not typically free from all human influence.

Today, in the wild, apes face the impact of the Anthropocene. Their once expansive historical geographic ranges and EEAs are therefore much reduced in size and changed in constitution. The degree and intensity of human impact has a direct influence on the social and ecological environment of wild primates and is shown to vary across long-term ape field sites (Hockings et al., 2015). Moreover, ape field studies are impacted by the presence of research itself, especially when the method involves habituation of individuals to frequent human observation. Habituation is still considered the gold standard for ape behavioural research, although it entails many risks including the introduction of human pathogens, increased vulnerability to hunting, and changes to behaviour (Allan et al., 2020; Gruen et al., 2013; Hansen et al., 2022). With respect to chimpanzees for example, some habituated communities have been reduced to small group sizes due to a combination of anthropogenic pressures (e.g. Bossou: Langlitz, 2020; Taï: Patrono & Leendertz, 2019; and Mahale: Kaur & Singh, 2008). Importantly, we know that these environmental changes are affecting the loss of ape behavioural and cultural diversity (Kühl et al., 2019). Although some apes are also exhibiting new behaviours as they adapt to human-dominated habitats, particularly chimpanzees (Hockings et al., 2015), the repercussions and consequences of these behaviours on the long-term viability of these populations is not yet well understood (Gilbert & Kalan, 2025). The tough question therefore is whether these affected populations should still be considered ecologically valid. Importantly, some field settings that are currently presented as ‘wild’ may produce data that is unrepresentative at the species-level and/or of apes in their EEAs. On the other hand, it may be argued that this situation increases the comparability of current captive and (typical) wild ape data.

It is perhaps misleading then to characterise all wild populations under a single – ‘wild’ term. In reference to Bossou chimpanzees for example, primatologist Osamu Sakuru remarked, ‘Yes, they were wild. but their environment was quite artificial’ (quoted in Langlitz, 2020, p. 242). Human contact and interactions therefore not only exist in captivity but also exist in the wild. This could potentially matter. Some scholars argue that intensive human contact or rearing in human environments actually *enhances* ape cognition (via so-called enculturation; Tomasello et al., 1993). It is possible that smaller effects may result from even minimal human interaction (McGrew & Tutin, 1978). These may be potential factors in the so-called captivity effect – the label given to cases in which captive apes seem to behaviourally outperform wild apes (Forss et al., 2015). With rising anthropogenic pressure, the potential for enculturation effects in wild populations may become even more relevant in the near future. Complicating matters further, human enculturation should not be expected to enhance all aspects of cognition, some may instead be diminished.

Some behaviours observed in the wild are often not possible or feasible in most captive settings, such as border patrols, hunting, predator mobbing. However, there are also behaviours that (to the best of our knowledge) appear in some captive settings that are not observed anywhere in the wild (to date), including distressing (e.g. social hair plucking in bonobos; Brand & Marchant, 2019) but also positive (for the apes) behaviours such as increased tool-use capabilities (e.g. chimpanzees hoarding stones as projectile weapons, Osvath, 2009 and greater rates of tool-use in bonobos, Gruber & Clay, 2016) and potential socio-cultural traits (e.g. placing objects in their ears, van Leeuwen, Cronin, et al., 2014). Hence wild and captive conditions may sometimes enable different behaviours. Or, as Jane Goodall (Van Lawick-Goodall, 1971) stated: ‘[captive] behavior could occur in the wild too, given the right environmental stimulus’. Therefore, even though wild ape habitats are typically more complex and diverse than captive settings, there is substantial variability among wild ape environments as well as captive housing conditions (e.g. sanctuaries versus zoos). Indeed many sanctuaries make efforts to incorporate natural environments, natural food items and more choice into the daily activities of apes where possible (Ross & Leinwand, 2020). We should therefore avoid falsely dichotomising all wild ape habitats and captive settings as one kind or the other.

Classifying entire research settings as valid or invalid without looking at the details is generally problematic. An empirically guided assessment of the relative effects of social and ecological environments (including food availability, perceived predation risk, etc.), and their interactions, across ontogeny for their potential implications on the research questions being asked seems useful (e.g. Fröhlich et al., 2021, 2024 and compare Leavens et al., 2010). There is a recent initiative called the ‘Ape Research Index’ which aims to tackle some of these questions (Bandini & Forss, 2023), complementing research into similar questions such as impacts of trauma (see above). More research will be needed to shed light on the many complex and interactive ways in which ape ontogeny affects their behaviour and cognition to result in group-level effects, such as ape culture (e.g. captive conditions, S. Forss et al., 2020; species differences, Forss et al., 2016). However, irrespective of where one works, we can probably all agree that what should be avoided is to take traumatised or deprived apes’ data as the *upper* limit to their behaviour and cognition. Personally, and pragmatically, we believe that of highest interest should be detections (if they exist) of reliable differences between *most* wild and *most* captive apes that prove larger than the natural differences that are observed across ape individuals and populations in *either* the wild or in captivity. Imagine one finds major differences between captive and wild apes where the former show higher tool making abilities than the other, and where these differences go significantly beyond or below the most extreme levels seen anywhere in the wild (or *vice versa*). We argue that this would be a more important result than finding any difference between a *single* wild and a *single* captive study. Relatedly, if wild apes were to be found to differ in profound ways from each other (including across populations), then this, too, would be of heightened interest. Note however, that wild-ape researchers would then run even more profoundly into the problem of what ‘wild ape behaviour’ or ‘wild ape cognition’ could even mean when used in a general sense (Leavens et al., 2010).

### 2.2. Double standard for (nonhuman) ape cultures?

There has been a longstanding critique from the onset of the ape culture wars that the criteria applied to nonhuman animals to demonstrate evidence for culture is not itself applied to human cultures. Often, these standards related to evidence for special underlying mechanisms. The late Christophe Boesch was one of the leading voices of this critique, referring to a double standard in culture research where, ‘No one seems to require human cultural differences to be acquired only through imitation or teaching’ (Boesch, 2001). This is not to imply that there is any doubt in the minds of most wild-ape researchers that humans are the most culturally-endowed primates on this planet. Rather, that if we are truly interested in the evolution of human culture then it would be fair to apply the *same* criteria to both humans and apes. The problem of course is that for humans, many take it for granted that culture – including any of its proposed mechanisms – is at play, while for other animals, including our closest living relatives, the default assumption is that this needs to be first proven.

This double standard critique plagued the ape culture wars for some time, particularly during the tenure of Boesch and Tomasello as joint directors of the Max Planck Institute of Evolutionary Anthropology back in the late 1990s and early 2000s (Boesch & Tomasello, 1998). During this time, Tomasello and his team often tested human children and great apes with similar behavioural paradigms, targeting various abilities including specific social learning abilities that may be restricted to – or at least especially pronounced in – humans over apes. These experiments seemed to have frustrated many wild-ape researchers, most notably Boesch, who questioned the experimental validity of these comparisons (as well as for ethical reasons: Boesch, 2012, pp. 41–44 and 192–200). The details of these debates are beyond current scope. However, note that these comparative experiments were intended to try to level the playing field between apes and humans and to address the double standard critique. Here, the intention was to subject humans to the same methodological and theoretical standards as apes, before concluding for or against the presence of certain abilities. Humans (here: children), just like apes, likewise had to prove that they were able to socially learn in specific ways (at the time, the focus was imitation, specifically action-copying, and joint attention), before it was permissible that these mechanisms could be linked to supposed special types of human culture. Similarly, humans would have to meaningfully surpass ape performances, or else any stated difference in mechanisms would have to be upended.

**Figure 2.**
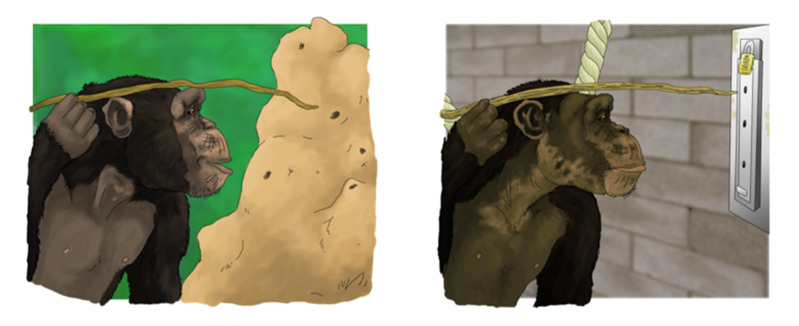
An example of how a wild-ape cultural behaviour, here termite fishing, is translated for captive-ape experiments. Note the differences in the environments and tasks. (*credit William Daniel Snyder, CC BY-SA 4.0*).

However, part of the double standard critique that Boesch emphasised was that the direct comparisons of apes and humans were done inappropriately, putting apes at a systematic disadvantage. Here, the idea that captive apes are unrepresentative as compared to wild apes once again featured heavily (see above) but also that testing apes in these experimental paradigms designed primarily for human children (e.g. by using human demonstrators even in the case of apes), put apes at an unfair disadvantage (Boesch, 2007, 2012). Another critique was the lack of physical separation of humans (parent and child) during testing (Clark et al., 2019), and that the chimpanzees tested by Tomasello’s team had a deprived and traumatised background as they originally stemmed from a medical facility (Boesch, 2012). Subsequently, some captive-ape researchers aimed to test these and related critiques via targeted experimental methods, often taking into account data from the wild (Figure 2).

When additional methods were applied and wild ape data incorporated, researchers found no clear signal for special types of social learning in captive ape studies, such as action-copying (Motes-Rodrigo & Tennie, 2021). Likewise, using trained *ape* demonstrators (compare Langlitz, 2020, p. 139) also did not lead to the occurrence of these specific social learning mechanisms in apes (Tennie et al., 2012), and neither did ‘behind bars’ testing prevent children from using these social learning abilities (Tennie et al., 2009). Contra the claim that the biomedical background of the Leipzig Zoo chimpanzees made them especially deprived (Boesch, 2012), the other ape species in Leipzig (who *lacked* this specific background), still did not show these abilities (Neadle et al.; Tennie et al., 2009), nor did sanctuary chimpanzees (Tennie et al., 2012). Across these studies, the sex of the demonstrator, using a physical or social task, or even having mothers as demonstrators (Tomasello et al., 1997) was shown not to matter. In a recent paper, Boesch and colleagues (Boesch et al., 2020) acknowledged that captive apes appear to lack in some types of social learning. However, many of the results deriving from such targeted experiments are often neglected by wild-ape researchers, even though the studies supporting these views now include wild ape data (Acerbi et al., 2022; Motes-Rodrigo & Tennie, 2021). This is not to say that the question of whether apes spontaneously use certain social learning mechanisms like know-how copying is considered answered (Tennie, 2023; Whiten, 2022). The debate persists as new research from both wild and captive apes continues to emerge (Boesch et al., 2020; Koops et al., 2022; Koops, Arandjelovic, et al., 2023; Neadle et al.; Pope et al., 2018; Tennie & Call, 2023, 2024) and as the validity of some existing studies (Horner & Whiten, 2005) as tests for particular social learning abilities continues to be debated (Clay & Tennie, 2018).

### 2.3. Moving goal posts in the ape culture war

Since the 1990s, when the ape culture wars can be considered at their peak, some wild-ape researchers perceive the scientific debate on ape cultures as one in which captive-ape researchers have continuously moved the goal posts for satisfying their definition(s) of culture, often seemingly in response to data published on wild apes. In particular, some wild-ape researchers argue that the requirements to demonstrate ape culture keep changing, becoming more complex and nuanced (i.e. social learning yes, but which *types* of social learning) as captive-ape researchers put greater emphasis on details and mechanisms rather than to accept behavioural variation between populations and the presence of social learning *per se* as settling the matter. This may sometimes be perceived by wild-ape researchers as a strategy by captive-ape researchers to emphasise the *differences* between humans and apes, rather than acknowledging the *similarities* (see below on ‘anthropodenial’). It is our belief that these perceptions are still at the centre of the rift between many wild-ape and captive-ape researchers, even though the call for a differentiation between social learning mechanisms was made since Yerkes (1916) and Köhler (1925), and even though some wild-ape researchers likewise put additional requirements on ape culture, such as non-subsistence (McGrew & Tutin, 1978; see also Langlitz, 2020, p. 58) or multiple traditions (van Schaik et al., 2003).

However, what is easily overlooked is progress. One differentiation of the kind we just mentioned has actually been surpassed by combining two types of social learning in the debate that, in the 1990s, were still kept apart. These two mechanisms are imitation (roughly: action copying) and emulation (roughly: results copying, i.e. changes in the environment) – which are now *both* widely regarded as potentially capable of enabling human-like culture to develop (Caldwell & Millen, 2009). Yet, at the same time some called for ape evidence for copying *beyond a level* principally achievable by motivated, typically developed individuals in the absence of cultural models (Tennie et al., 2020, 2009). Evolving variants of this latter approach continue to be criticised for goal post shifting (Koops, Biro, et al., 2023; Whiten, 2022; see also responses of Tennie & Call, 2023, 2024). Yet, changes in approaches should not be problematic *per se* given that the aim for hypotheses and theories in the scientific method is to be continuously evaluated and updated in the light of changing evidence. It would perhaps be more problematic, if no changes occurred.

## 3. Critiques from captive to wild-ape research

### 3.1. Relative lack of interrater reliability measures in the field

Time and time again, research has shown that individual judgements are fallible (Senders & Moray, 2020). Likewise, perfect coders do not exist (whether in captive or wild settings). This makes it necessary to provide reliability measures for data. However, there is a lack of specific kinds of reliability coding relevant for ape culture or behavioural variation studies in the wild. In many cases, what does happen is that interrater reliability of live coding is done initially to train and/or assess coders yet these data are typically not published, making it difficult to know how reliable observations truly are across individuals. Moreover, to the best of our knowledge, the criteria for the training and for the assessments have never been formally evaluated, posing a risk for creating and stabilising observer ‘group drift’ – research group’s specific biases in coding (Girard & Cohn, 2016).

When examining recent publications for their reporting of interobserver reliability coding in both wild and captive zoo studies, we note that interrater reliability is more often missing from wild-ape studies. When reliability coding has been reported for wild-ape research using video data, we observe a high degree of reliability variability across behaviours (Boesch et al., 2020; Fröhlich et al., 2016; Kalan et al., 2019; McCarthy et al., 2019). The expected result of such coding is similar to data coding in captive work: sometimes, the coded data (say, category *X* of the coding) will be sufficiently reliable, sometimes it will not be. Crucially, only the former type of data should be analysed. Field situations widely differ, and coders not only differ in their ability to code, they also differ in their ability to code different types of data and they may also differ in these abilities across time, which is where *intra*rater reliability becomes significant. After passing initial training, there is often the assumption that coders remain reliable henceforth, and that their data is reliable for all kinds of behaviours. Both of these assumptions are questionable on theoretical (Girard & Cohn, 2016), and empirical grounds. For example, rare behaviours may often be missed even by trained coders (Wark et al., 2021). While some categories may remain reliable over time (at least under coding test situations), others may deteriorate after mere months (Stubsjøen et al., 2022), while other behaviours may not have been part of initial training. In sum, reliability is potentially quite low for some wild-ape studies (compare Langlitz, 2020). There are notable exceptions, such as ape gestural research, where in both the wild and in captivity reliability of coding are reported for each study (Pika & Liebal, 2012). Ideally, field sites would continuously monitor (at regular intervals) and report reliability across (inter) and within (intra) observers, especially when new behaviours are being targeted for data. We observe that many captive-ape studies also appear to lack intrarater reliability, which may reflect shorter lengths in study durations compared to wild-ape studies.

Why is reliability so important? Without good measures of observer reliability any validity derived from these observations are questionable. Indeed, it would be difficult for most captive-ape research to be published without interrater reliability coding done for each study, and without this coding meeting at least minimal quality criteria. Perhaps this is because of carry-over effects of the adjacent field of developmental psychology, where interrater reliability measures are very often reported (see above). This is not to say that captive-ape researcher reliability coding does not face any issues, as we have already mentioned that intrarater reliability is often lacking here too. There are also additional issues with coders-degrees-of-freedom (Neadle et al., 2025) which may be more prevalent in captive-ape studies, but overall, in our experience and observations, interrater reliability coding is more often reported in captive-ape studies.

Reliable data coding is far from trivial (Graham et al., 2021), not only in general, but specifically with regards to behaviour, and especially under field conditions when observing wild animals that have a repertoire of hundreds or thousands of behaviours like apes. Furthermore, imagine a dark forest with lots of leaf cover, maybe some rain, too. Imagine fast-moving apes, or individuals partially hidden behind a tree and being some distance away. Further imagine a situation in which no video recording is being made at all, but all coding must be done live. In these types of situations, a single coder has only ever one attempt to get the coding right. This is hard to do, and risks observations becoming obsolete when this original observer leaves the project. These imperfections leave room for biases to emerge and for deviations to occur from ‘real values’. In our experience, these situations are the norm rather than the exception at some ape field sites which may contribute the majority of direct observations used in culture research. The predictable outcome of such challenging situations is poor coding reliability, if it were measured. In most cases, the current practice of single-person ‘live-coding’ in the absence of video records and in the absence of additional similarly focused live-coders present (e.g. two-person live interrater reliability coding) means that reliability issues under field conditions are often neglected.

One exception are studies in the wild which, alone or in addition to live coding, collect (at least some) video recordings. In such cases, it is possible to determine reliability measures from multiple coding of these videos and/or from comparisons of video coding to live coding (when available). The other exceptions are field studies for which multiple live codings occur simultaneously (typically two-person live coding; e.g. Perry et al., 2008). If, in addition, these live codings are on the same animals at the same time, and use the same criteria, interobserver reliability of live-coding can be derived and even replicated (a standard we recommend). Video data, both from habituated (Fröhlich et al., 2021) and unhabituated apes (Boesch et al., 2020; Kalan et al., 2020), is particularly powerful for ape culture research, not least because it can be used for reliability tests and to clearly demonstrate and digitally ‘conserve’ ape behaviours (see above). Video data will also likely serve as time-capsules for future researchers. Videos of cultural behaviours, or at least samples of those behaviours, placed under open access would greatly benefit ape culture research to avoid misunderstandings, particularly with observational data (e.g. open access video library accompanying [Boesch et al., 2020]; https://www.eva.mpg.de/primat/staff/boesch/termite-fishing-video-library).

Of course, even when such things are done, issues may remain. First, video recordings are of poorer quality than live views. Second, the video recordings would need to actually be used for reliability coding. Third, live co-coding is labour-expensive, and difficult to coordinate – and if two (or more) people live code together, it risks unreported, informal comparisons and data alignments between these coders during data collection (which would artificially inflate interrater reliability and likely go unreported). So, issues remain, but they should not outweigh the benefits of reliability testing of data. Yes, this will add additional workload to an already intense data collection method, especially in the field, but we see no alternative. Reliability is important and cannot be skipped. Computerised coding from video – with at least the possibility of reliability coding achievable in this new automated way – could help to save time (Gammelgård et al., 2024). Lastly, data proven to be reliable does not mean that it is absent any bias. Reliability coding is not suitable for removing bias (Freeberg et al., 2024). Observer bias is a different problem altogether (see section D below). In summary, we think the trust in wild-ape data would substantially improve, and increase in its power to convince sceptics, if reliability measures were more commonly provided.

### 3.2. Lack of control conditions in the wild

Captive-ape researchers have often argued that low-level explanations for seemingly complex behaviours may be more parsimonious than invoking higher-level cognition (Tomasello et al., 2012; but see also Mine et al., 2022). This has also been true with regards to social learning mechanisms whereby confounding effects of widespread mechanisms such as stimulus or local enhancement (social learning of ‘know-what’ and ‘know-where’; Tennie et al., 2020) is often difficult to rule out completely in the wild, not least because of the limited ability to control the environment but also due to ethical constraints. Field conditions make it difficult to test and target alternative hypotheses or mechanisms in wild populations. Here, research in captivity typically has much more freedom and experimental control over presentation of stimuli and manipulation of the environment (including the social environment).

Everything else being equal, it is much easier to do experiments in captivity than in the field. The main critique is therefore the *relative* lack of experimental control in the field. Lacking experimental control, it is sometimes difficult to pinpoint with precision the validity of explanations, especially of rare behaviours in complex situations. Note that this does not mean that all captive work is well-controlled, but merely that it often is better controlled than wild-ape research. To those used to heavily controlled data, pure observational data might seem less convincing, because it may *look* inherently uncontrolled. However, a lack of experimental control does not equate with a lack of *any* control. Quite simply, systematic data collection can eventually lead to data richness that allows to tease apart causality structures even in complex contexts, especially with the advent of modern statistical methods. What is key here is that such data collection expands across long time periods to allow the capture of the outcomes of multiple rare situations. Wild-ape researchers must therefore patiently await the recording of many such ‘natural experiment’ situations, in which the details of a particular context are coded, along with the behaviour of those apes in that context (e.g. waiting until a group of chimps happen to get close to a rare type of fruiting tree). Wild-ape researchers then ‘merely’ have to wait until enough of these situations have been observed and recorded. This strategy works if these situations arise with at least a sufficient frequency. Very rare events remain difficult to disentangle in this way. In some cases, situations may occur so rarely in natural settings as to exceed the careers of single researchers seeking to collect enough cases.

However, we note there is some progress. One way to massively increase sample size of even rare events is by expanding the number of populations under study (e.g. Pan African Programme: the Cultured Chimpanzee; Kühl et al., 2019) by using remote technologies such as camera-traps to collect video observations that allow for reliability coding (Kalan et al., 2019; McCarthy et al., 2019).

Another way is to run experiments in the wild while being mindful of ethical concerns; although some populations and tests may be better suited for this than others. These experiments can take various forms, such as presenting models of predators, pictures of conspecifics, or food availability manipulations (Fischer, 2022). Tetsuro Matsuzawa, for example, created an ‘outdoor laboratory’ for ape tool use at his field site Bossou (Langlitz, 2020, p. 240). Others have followed in a similar vein (Gruber et al., 2009; Koops et al., 2022). Indeed, in at least some domains, such as vocal communication, (playback) experiments in the field can be considered commonplace (Crockford et al., 2017; Zuberbühler, 2014).

Field experiments face a couple of problems. First, they are generally more constrained and limited than what is possible to test and rule out in captivity. Second, some experimental controls can be perceived as introducing artificial elements (Koops, Biro, et al., 2023), so their implementation can invalidate results. There is more nuance however to this which should be considered. For example, placing a locally available nut ‘in a controlled way’ into a chimpanzee group’s environment, without ape onlookers, is an artificial intervention by definition (the human placement simply makes it so). Yet it does not strike us as an uninformative way to collect data on reaction to these nuts, as apes can encounter them in their environment. Careful artificial experiments can mimic natural experimental contexts.

A potential issue with experimental settings (in the wild and in captivity), even if they successfully mimic natural (but rare) situations, is that they inflate the frequency of those situations, effectively turning naturally rare events into artificially common ones. This frequency increase could potentially influence behavioural outcomes. For example, via desensitisation or via increased chance of pattern recognition. Depending on the research question, these effects could influence the validity of derived claims. This should be investigated in future studies but in the meanwhile assessing long-term baseline rates for behaviours has been proposed (Bandini, 2021).

### 3.3. The investment fallacy is especially pronounced in the wild

Another reason why wild-ape researchers may feel dismissed by captive-ape researchers is that the former typically must work very hard to retrieve any data from the field. It is understandable that particularly hard-earned field data – involving physical, social, psychological, and physiological difficulties – may be perceived as more valuable by those who worked hard for it. Relatedly, there seems to be a general romanticism and normativity of ape field data collection. This means that, *on average*, the effort justification (a kind of investment fallacy) will typically affect wild-ape researchers harder than it does captive-ape researchers. This conclusion follows findings on human biases in economics, psychology, and philosophy (Celniker et al., 2023; Inzlicht et al., 2018). For this and other reasons, the viewpoint of captive-ape researchers is (this is our impression; systematic studies are not yet done) that the epistemological value of wild-ape data is sometimes insufficient for making some of the claims that wild-ape researchers wish to derive from it.

In our experience, this situation is not commonly acknowledged by wild-ape researchers. Yet two things can be true at the same time. Wild-ape data is clearly special in some ways (see Part B1). Yet it does not follow that wild-ape data holds special epistemological weight because of its hard-won nature. After retracting the effects of the investment fallacy, such data must be judged via criteria that have nothing to do with how difficult it was to collect the data.

## 4. Metacritique of both captive and wild-ape research

Interest in ape behaviour and cognition remains high, both academically and on a societal level. We believe that this situation calls for best-practice science both in captive- and wild-ape research, as well as charitable interactions between these fields. Below, we focus on both scientific and political issues that highlight shared problems across ape culture research.

### 4.1. Systematic differences in observer bias

There is a museum in Japan with a display of several hundred stones resembling human faces (the Chichibu Museum of Rare Stones). This charming display caters to the well-known human bias to perceive illusory images (called pareidolia). What makes these stones look like faces is entirely in the eye of the beholder. A biased observer can perceive things that do not exist. When, as in the case of the stone museum, this bias is embraced and highlighted, the effect can be positive, pleasing and aesthetic. However, observer bias is an obvious hindrance in the pursuit of scientific endeavours (one of many such hindrances; compare Langlitz, 2020, pp. 48, 66, 128, 129).

Observer bias exists in ape studies too, just like it exists everywhere (Tuyttens et al., 2014). More importantly perhaps, observer bias in ape studies might be systematically and differentially amplified via cultural differences between captive- and wild-ape researchers themselves. At some points of their careers, most (not all) ape researchers effectively *sort themselves* into either becoming captive or wild-ape researchers (compare Leifeld, 2018). This sorting may be based on pre-existing biases (e.g. different perceptions of these two types of research gained in classes or from media). Once sorted, the result is differentiated social and academic communities which may exert additional cultural influences on observer biases (e.g. Sulik et al., 2025). In this second case, we would expect these biases to solidify and maybe even amplify. The outcomes may then, in various ways, feed forward into the next selection of researchers (*and so on*). In the following, we describe tendencies in our respective research fields that we have personally experienced or observed, and may be *potentially* contributing to differences in observer bias.

One tendency we observe is that captive-ape researchers are often more cautious to accept positive evidence of cultural phenomena, whereas wild-ape researchers are often more enthusiastic. Given that captive-ape researchers report on the impact of observer bias more often than fieldworkers (see above), they frequently ‘explain away’ fieldworkers’ claims by reference to observer bias. However, captive-ape researchers are not bias-free, and there can also be biases and preferences by the apes themselves when interacting with certain human experimenters, further influencing observer bias (Schubiger et al., 2020). A special variant of observer bias was highlighted by the late Frans de Waal (a captive-ape researcher). de Waal noted that observer bias can also work *in reverse*, whereby observation ‘filtering’ may install *excess* filters, over and beyond what may be reasonable. De Waal termed this excess filtering ‘anthropodenial’ (de Waal, 1999). This phylogenetically informed idea was that in certain cases one can be too concerned with observer bias particularly when the observed is a closely related species. de Waal urged to lower these filters when studying apes.

We think de Waal had a good point here – a point related to the double standard critique above (see Section B2) – and the suggestion remains important. However, the result should not be a complete lack of filters. The very existence of biases is not in question, and the same is true for reasons to avoid these. Metaphorically speaking, we need to avoid situations in which stones *really* are seen to have faces in that Japanese museum. After all, even when human researchers look at human behaviour, they must do so critically. Moreover, when we look at the many diverse filters applied in fields concerned with observing humans (e.g. child development; Davis et al., 2021), we clearly see that our senses can mislead us, especially if the target of our observations are related to us (see the replication crisis in psychology; which is not even the first of its kind; Lakens, 2025). We need filters for observing apes, just like we need filters for observing humans (Langlitz, 2020, p. 77). The only viable path requires us to accept that both anthropomorphism and anthropodenial exist and that both need to be avoided.

### 4.2. A replicability crisis in ape culture research?

The 2010s saw the field of psychology being called out for a phenomenon known as the ‘replication crisis’. We do not wish to go into detail here, so let us simply say that this crisis has uncovered that psychology is prone to publishing claims that cannot be supported by follow-up studies. Yet, the replication crisis is likely to affect other fields as well (Baker, 2016) and it may only be a matter of time before the replication crisis hits primatology (Farrar et al., 2022). In some sense, this has already happened in captive-ape research (see so-called Hausergate; Miller, 2010). Likewise, wild-ape researchers are not exempt from this crisis. For example, reviews of primate communication literature, including both wild- and captive-ape research (Waller et al., 2013), and playback experiments more generally (Kroodsma et al., 2001), have detected issues in pseudoreplication. Studies in wild primate communication were particularly prone to suffering from pseudoreplication (Waller et al., 2013). Pseudoreplication is but one of many ways in which statistical errors thwart replication of studies, undermine published results, and propel the replicability crisis. There are many additional issues and pitfalls that may apply to ape cultural research, such as p-hacking, and small sample sizes (Stevens, 2017).

One reason why the replication crisis has not fully hit ape research (despite the clear issues with reliability described in Section C1) may have to do with the fact that replication attempts are generally rare in field primatology (Langlitz, 2020, p. 176). The issue is further complicated by the difficulties in openly sharing data from long-term wild-ape field sites (see below). Ideally, we should know what the replication rate overall really is in primatology (Farrar et al., 2022). Yet, the few cases where replications occurred and failed, should be cause for alarm (e.g. a captive replication failure; Motes-Rodrigo & Tennie, 2021). Some might argue that replication failure is no cause for concern because ape populations are so different from each other that a replicated finding is not to be expected. But note, if that were true, then we would be unable to generalise any data from ape populations to a species level.

Of course, we are not the first to say much of this (e.g. Langlitz, 2020). There are continuous efforts underway to make primatology better, and indeed issues like pseudoreplication mentioned above appear to be less prevalent in more recent communication research (Whitehouse et al., 2024). Yet, primatology in all its facets should strive for and embrace methodological advances and fail-proofs – in particular, those that have been developed in direct response to the replication crisis elsewhere. This includes Registered Reports and large-scale collaborations or consortia such as the ManyPrimates project (Primates et al., 2019) and ManyManys (https://manymanys.github.io/).

One important avenue to increase the validity of ape research is to promote greater exchange and collaboration between captive and wild-ape researchers. Experiments on apes in captivity can benefit from the expertise of fieldworkers (Koops, Biro, et al., 2023), while experiments conducted in the wild can similarly benefit from the expertise of captive-ape researchers. Here, collaboration, or at least consultation or communication, can improve methods, increase reliability and validity, and may provide opportunities for both sides to work together more often. To fully harness the power of both wild-ape and captive-ape research, we should encourage and support ape culture researchers to obtain training and experience in both sets of research conditions and methods. Our belief here is that researchers that are not strictly wild-ape researchers, nor strictly captive-ape researchers, but rather have expertise in both, may provide significant insights moving forward. Note however, that we do not wish to make any of these recommendations an absolute must – as doing so could foster power plays (e.g. via field/captive access restrictions), but also because this could introduce ableism and financial constraints. Instead, we highly encourage more cross-discipline collaboration and dialogue, where it is possible and feasible.

### 4.3. The politics of the ape (culture) wars

We have focussed thus far on the science of the ape (culture) wars but as may be expected in such situations, there are politics at work here, too. Primatology in general suffers from issues associated with parachute science, including but not limited to, inequity, racism, neocolonialism (Odeny & Bosurgi, 2022; Setchell et al., 2024), ethical concerns for ape and human co-inhabitation, moral obligations of researchers working in captive and wild settings, and ableism (Bezanson et al., 2013; Gruen et al., 2013; Hansen et al., 2022; Mitani et al., 2024; Riley & Bezanson, 2018; Ross et al., 2022). These and other issues remain significant and pressing, but there are additional political aspects currently underappreciated, in our view, for their effect on ape culture research.

Given the intense public and scientific interest in ape behaviour and cognition, perhaps especially ape cultures, this research domain has generated powerful scientific entities, institutions and individuals alike. With this power comes the danger of preventing scientific progress, even via questionable methods such as gatekeeping and gossip. These toxic practices result in the greatest harm to early career researchers, and those without strong ties to rich, predominantly western institutions, thereby also preventing diverse scientific voices from making impactful or continuing contributions to the ape (culture) debate.

Gatekeeping is not only true in scientific circles, but beyond. Most significantly the ape culture wars are relevant to current initiatives to use culture as an additional metric in guiding conservation in the wild. International organisations such as the UN and IUCN have been actively examining the myriad of ways in which culture can be leveraged as a conservation management tool to protect wild ape populations (Brakes et al., 2021; Kalan & Luncz, 2025). These organisations are relatively unconcerned with the major scientific debates surrounding the nature(s) of ape cultures. Rather, the focus is on the dire status of wild ape populations, all designated as Critically Endangered or Endangered. Cultural primatologists, working in both wild and captive settings, have drawn attention to the potential pitfalls that may arise if certain populations or even single cultural behaviours should be singled out or prioritised for conservation efforts based on cultural claims (Carvalho et al., 2022; Motes-Rodrigo & Tennie, 2021). Ape culture then, as perceived and judged by a small number of scientists, can now have real life or death consequences for wild apes. In fact, the conservation impact of ape culture illuminates why the ape culture wars have not dissipated given the now intense ethical and emotional consequences of this type of research. We may risk repeating the same mistake for apes as has already been done to some human populations: condemning them based on culturally derived judgements (Langlitz, 2020).

Another facet of the ape culture wars that has perhaps been underappreciated until recently is that much of what we know about ape cultures from the wild is due to the long-term field sites that have existed for decades despite numerous obstacles (consistent funding being one of them). The reason this is significant is because in the current scientific revolution for open science and increased transparency, this often requires making complete datasets readily available or even published alongside journal articles – which many PIs of long-term field research sites find problematic (Mills et al., 2015). Given the physical, mental, financial and emotional toll of wild-ape research, we observe that these issues are quite pressing for those who manage ape field sites. Wild-ape data is in many ways ‘like gold’, hard to get and therefore highly valuable (within limits, see Section C3); we must therefore find ways to do open science in fair and sustainable ways. The recent trend to use non-invasive technology to study unhabituated, and sometimes also habituated apes, has helped to create more open data with respect to wild populations (more data, quicker, and easier to share, etc., see Hansen et al., 2022 for a review).

We sympathise with PIs of long-term ape field sites who feel they are being cornered into a position to ‘give up’ their hard-earned longitudinal data for the sake of embracing one-size-fits-all publishing standards, making themselves potentially vulnerable to being ‘scooped’ by other researchers who mine their open datasets. Some say the risk for this is low (Evans, 2016), but in reality, ape data is hard-to-get and in high demand. Large ape data sets might attract what may be perceived as drifters by the original data producers. The issue of open data is further complicated when we consider that great apes live in countries located in the Global South where researchers from these countries face numerous obstacles in advancing in their careers. Hence if and when local scientists are involved in data collection, any appropriation of these data may further disadvantage these individuals. There is also the long-term issue of the exclusion of local scientists when it comes to wild-ape research (Langlitz, 2020; Setchell et al., 2024) which we think is slowly showing improvement, especially in regards to the conservation sector and large-scale collaborations (e.g. PanAf https://panafrican.eva.mpg.de/).

Overall, long-term field data availability represents a tricky issue, and as detailed in Mills et al. (2015) there may be a number of potential solutions (e.g. data embargoes, sharing data on a confidential basis) which better acknowledge the cost of long-term data collection. We would argue that funding agencies also need to be active in discussing solutions with field site managers which would hopefully lessen the strain of running a long-term wild-ape field site. In sum, there may need to be higher incentives for long-term research site PIs to make their data open access beyond publications alone, as well as reductions of risk, or at the very least systems that co-reward original authors when others use their data (e.g. see the APES Wiki initiative: https://www.iucngreatapes.org/apes-wiki).

## 5. Conclusion: recommendations moving forward

This paper has dealt specifically with ape culture, but as mentioned in the beginning, many of the critiques and concerns will apply to a broader range of research topics (e.g. cooperation) that have similar polarised debates and maybe even to other target species and genera. It is important to recognise that wild-ape researchers and captive-ape researchers, sometimes working in both settings (e.g. van Schaik, Matsuzawa), have *all* contributed to our current understanding of ape culture. Across the decades, both sides have contributed to improving ape research methods, in both the wild and in captivity, often learning from cross-disciplinary exchange (e.g. impacts of provisioning apes, the effects of enculturation, the carry-over of focal observation methods from psychology to primatology (Langlitz, 2020, p. 127) and from archaeology, too (Mercader et al., 2002). We therefore end this paper with a call for promoting open mindedness on both sides and for moving away from our biases and gatekeeping tendencies so that we may truly listen and learn from one another. Based on our assessment of the issues described in the previous sections, we highlight the following recommendations for researchers on both sides of the divide:

For wild-ape researchers:

- Make monitoring and reporting of interrater reliability measures standard practice and only use reliable data for analyses.
- Remain open to experimental manipulation. There can be naturalistic experiments. Of course, some experiments remain non-advisable, especially if they raise valid ethical concerns.
- Consult captive-ape research (and, where possible, captive-ape researchers) when conducting wild ape experimental research.
- Be aware of the investment fallacy and anthropomorphism.

For captive-ape researchers:

- Be open to the idea that there can be behavioural and cognitive differences associated with certain captive and wild settings, especially if apes are exposed long-term.
- Do not equate a lack of experimental control with a lack of any control.
- Consult wild-ape research (and, where possible, wild-ape researchers) when conducting captive-ape research, especially when you do observational studies.
- Be aware of anthropodenial.

For both:

- Consider the multifactorial, complex, and time-dependent influences on ape social learning and its implications for ape culture.
- Primatology’s replication crisis may be around the corner. Strive to replicate key findings (with reliability measures), publish Registered Reports and consider joining research consortia.
- Try to remain open-minded to the ‘other side’ and be charitable in your reading and discussions of other’s research.

In closing, we would like to highlight what we believe are emerging scientific directions in ape culture research that have the potential to unite captive-ape and wild-ape researchers by combining their various expertise in exciting new ways:

1. Examine cultural variation in a more nuanced, detailed way (e.g. Boesch et al., 2020) rather than solely focussing on presence/absence of behaviours. Ape techniques, styles, and ways of doing things on a finer resolution, may uncover hidden cultural variation. However, zooming into these details will require not only the use of reliability measures, but also especially good reliability, given increased coding difficulty when focusing in on details.
2. More research attention to detect behaviours and behavioural patterns that are locally unique, rare, or locally restricted especially in the wild, given these may be – everything else being equal – the best-candidates for human-like cultural mechanisms (Motes-Rodrigo & Tennie, 2021; Whiten et al., 2001). Plus, to avoid potentially misleading terms such as ‘group specific’, when the intended meaning is ‘not universally present in *all* populations’. For example, group-specific could then define behaviours that are unique to a *single* culturally connected group of apes (either a population or a population cluster, present today or in the past).
3. Increase research attention to the social, physical and ecological factors that can effectively deprive (even traumatise) individual apes – or conversely, stimulate them – in both captive and wild settings. These effects may lead to differences in cognition, particularly social learning type(s) and/or their relative power, as they relate to cultural capacity. In that same vein, it is important to investigate how negative effects can be reversed or at least improved under the right conditions. We also need to examine how strong and long-lasting the effects of human interventions may be (e.g. rearing conditions, experimenter preferences, etc.).
4. Support training and expertise of ape culture researchers (including ape range nationals) in both captive and wild-ape research methods and outcomes, to cultivate a field that regularly engages with cross-discipline dialogue, charitable interpretations of each other’s work, metascience, and thoughtful consideration for the different types of data that can get us closer to understanding the nature of ape and human culture.

For the sake of all apes, captive and wild, let us foster greater respect for one another’s research and find ways to bridge the gaps that alienate us. After all, scientific ingenuity benefits those who work (or at least discuss) together.

## Data Availability

There are no data, resources, or code associated with this article.
